# Uncertainty Meets Disordered Eating and Body Image: A Transdiagnostic Network Study Across Depressive, Anxiety and Anorexia Nervosa Symptoms Including a Control Group

**DOI:** 10.3390/nu18091370

**Published:** 2026-04-27

**Authors:** Roser Granero, Isabel Krug, Litza Kiropoulos

**Affiliations:** 1 Department of Psychobiology and Methodology, Universitat Autònoma de Barcelona, 08193 Barcelona, Spain; 2 Ciber Fisiopatología Obesidad y Nutrición (CIBERobn), Instituto Salud Carlos III, 28029 Madrid, Spain; 3 Group of Psychoneurobiology of Eating and Addictive Behaviours, Neuroscience Program, Bellvitge Biomedical Research Institute (IDIBELL), 08908 Barcelona, Spain; 4 Melbourne School of Psychological Sciences, University of Melbourne, Melbourne, VIC 3010, Australia; isabel.krug@unimelb.edu.au (I.K.); litzak@unimelb.edu.au (L.K.)

**Keywords:** uncertainty, anorexia nervosa, anxiety, major depression, network analysis

## Abstract

Background and objectives: Intolerance of uncertainty (IU) is a well-established transdiagnostic process in anxiety (ANX) and major depressive disorder (MDD), and has been increasingly implicated in anorexia nervosa (AN). However, most previous research including patients with AN has relied on total or subscale scores from eating disorder measures, which obscures how specific eating attitudes and body dissatisfaction symptoms relate to distinct facets of IU. The primary objective of the present study was to characterize item-level networks linking eating attitudes, body dissatisfaction, and IU in a pooled clinical mental health sample, alongside a control group (CG). Methods: Data were drawn from a sample including individuals with symptoms related to AN (*N* = 105), MDD (*N* = 97), and ANX (*N* = 240), a comorbid group (*N* = 84) with symptoms of two or more of these conditions, and a CG (*N* = 842). Separate item-level networks were estimated for clinical and control groups, and network structure and centrality indices were compared. Results: Network analyses revealed distinct organizational patterns between the clinical and control subsamples. Although both networks showed identical diameters, the clinical network exhibited a shorter average path length and higher clustering, indicating stronger local connectivity, whereas the control network showed higher modularity. In the clinical subsample, nodes related to binge eating, post-eating guilt, and IU emerged as the most central and acted as key connectors between clusters. In contrast, the control network displayed a more distributed centrality pattern, suggesting a more integrated and homogeneous network organization. Conclusions: This study provides new evidence to refine our understanding of how IU relates to eating attitudes and body dissatisfaction across diagnostic mental health boundaries. Identifying highly influential psychopathological symptoms across eating, mood, and anxiety disorders, as well as bridge nodes linking these mental health domains, is important for understanding transdiagnostic symptom dynamics. These insights may inform the development of more sensitive screening and diagnostic tools, as well as targeted intervention points to support more personalized and mechanism-focused treatments.

## 1. Introduction

Intolerance of uncertainty (IU) refers to a dispositional tendency to perceive uncertain situations as threatening and to respond with heightened distress, negative affect, and efforts to reduce or avoid uncertainty [1]. The internal structure of this complex construct remains unclear; however, network studies have shown that three distinct communities can be identified in both clinical and population-based samples, corresponding to negative beliefs about uncertainty, behavioral responses to uncertainty, and emotional responses to uncertainty [2]. It is known that this construct is implicated in the modulation of neural and psychophysiological responses to uncertainty, as well as in action tendencies and subjective emotional experience [3]: elevated IU is associated with heightened physiological reactivity, avoidance-oriented behavioral patterns, and increased self-reported negative affect, indicating its influence across multiple levels of emotional responding.

Initially, IU was conceptualized within cognitive–behavioral models of worry and generalized anxiety disorder [4,5,6], but it has since been incorporated into broader transdiagnostic and hierarchical frameworks of emotional disorders [7,8,9]. These models propose that IU contributes to psychopathology by amplifying threat appraisals, undermining adaptive emotion regulation, and promoting maladaptive coping strategies such as avoidance, reassurance seeking, and excessive control [10,11,12,13].

Consistent with these accounts, a substantial body of empirical evidence has observed elevated levels of IU across anxiety disorders (ANX), including generalized anxiety disorder (GAD), social anxiety disorder (SAD), panic disorder (PD), and obsessive–compulsive disorder), and these increased levels of IU have been found to be associated with greater symptom severity, chronicity, and functional impairment [14,15]. Studies have also observed the relevance of targeting IU in treatment for ANX [16,17,18]. Importantly, heightened IU is also observed in major depressive disorder (MDD) and has been linked to rumination, hopelessness, and negative cognitive biases [19,20,21]. Longitudinal and experimental studies further suggest that IU predicts the onset and maintenance of anxiety and depressive symptoms and mediates the impact of stress on emotional distress [22,23]. Together, these findings support the conceptualisation of IU as a robust transdiagnostic vulnerability factor underlying anxiety and depression, providing a strong empirical foundation for examining its relevance in other forms of psychopathology.

Growing evidence suggests that IU also plays a meaningful role in eating disorders [24,25,26], particularly anorexia nervosa (AN) [27]. Individuals with AN frequently exhibit elevated rigidity, cognitive inflexibility, and a pronounced need for predictability and control [28,29], characteristics that closely align with core features of IU [30,31]. Empirical studies consistently report higher levels of IU in individuals with AN compared to healthy controls, with IU being associated with eating disorder severity, dietary restraint, and compulsive weight- and shape-related behaviors. From a functional perspective, controlling food intake, body weight, and shape may serve as strategies to reduce perceived uncertainty and associated emotional distress, thereby reinforcing restrictive eating patterns [32]. Heightened IU has been shown to exacerbate anxiety related to eating, weight change, and bodily sensations, contributing to the maintenance of restrictive behaviors even in the presence of adverse physical and psychological consequences [24,25]. Importantly, from a nutritional standpoint, elevated levels of IU may promote rigid and rule-based eating behaviors by rendering ambiguous internal states (such as hunger, satiety, or postprandial sensations) aversive and difficult to tolerate [28,33,34]. These patterns often manifest as restrictive food choices, avoidance of specific food groups, strict meal timing, or excessive caloric monitoring, with direct implications for dietary adequacy and variability in eating disorders such as AN. Framing IU as a transdiagnostic mechanism influencing eating behavior thus provides a conceptual bridge between psychological vulnerability and nutritionally relevant outcomes, highlighting the potential value of interventions targeting both dietary flexibility and tolerance of uncertainty.

In addition, body dissatisfaction represents a core and transdiagnostic feature of eating disorder psychopathology [35] and is particularly prominent in AN, where a persistent negative evaluation of body shape and weight is maintained despite severe underweight [36]. In this population, body dissatisfaction functions as a central motivational factor sustaining restrictive eating behaviors [37], including prolonged energy restriction, rigid dietary rules, and systematic avoidance of foods perceived as calorically dense or nutritionally “unsafe.” These maladaptive eating patterns directly compromise dietary quality and result in insufficient intake of essential macro- and micronutrients, contributing to the development and maintenance of malnutrition [38,39]. Beyond weight loss, chronic nutritional inadequacy in AN is associated with adaptive metabolic changes, endocrine alterations, and multisystem medical complications [40]. Consistent with this, elevated body dissatisfaction has been linked to greater eating disorder severity, poorer treatment response, and increased risk of relapse [41,42], highlighting its role as a key psychological mechanism through which disordered eating behaviors negatively impact nutritional status and long-term health outcomes.

Body dissatisfaction has also been consistently associated with affective and anxiety-related psychopathology [43,44]. In MDD, negative body-related self-evaluations are closely intertwined with core depressive features such as low self-esteem, feelings of worthlessness, and pervasive negative self-schemas [45,46], contributing to the maintenance and severity of depressive symptomatology. Individuals with elevated body dissatisfaction are more likely to experience persistent rumination, social withdrawal, and diminished engagement in health-promoting behaviors, including exercise and adequate nutrition, all of which may exacerbate depressive outcomes and increase risk for chronicity [47,48]. Moreover, body dissatisfaction can amplify cognitive distortions and negative affect [49], creating a feedback loop in which depressive symptoms and self-critical body perceptions mutually reinforce each other.

Similarly, in patients with ANX, body dissatisfaction has been linked to heightened self-focused attention, fear of negative evaluation, and avoidance of social or body-exposing situations [50]. This is particularly evident in conditions characterized by elevated social threat sensitivity, such as social anxiety disorder, where appearance-related concerns intensify anxious arousal, vigilance, and safety behaviors [51]. Physiologically, persistent anxious symptoms in the context of body dissatisfaction may exacerbate dysregulation of stress-response systems, including hypothalamic–pituitary–adrenal axis activity and autonomic arousal, contributing to somatic symptoms and impairing adaptive functioning [52,53]. Socially, individuals with high body dissatisfaction often report increased peer victimization, stigmatization, and difficulties in interpersonal relationships, which may further reinforce both anxiety and depressive symptomatology [54].

Importantly, empirical evidence also suggests that body dissatisfaction may be closely intertwined with IU [55,56,57]. Heightened sensitivity to uncertainty may amplify concerns about bodily change, weight fluctuation, and loss of control over physical appearance, fostering rigid and negatively biased body evaluations [58,59]. In this context, body dissatisfaction may function not only as a central symptom of eating pathology but also as a potential bridge linking uncertainty-related distress to restrictive eating and control-oriented behaviors. Despite its clinical relevance, the specific ways in which individual body dissatisfaction experiences connect to distinct facets of IU remain poorly understood, highlighting the need for symptom-level approaches capable of disentangling these associations.

To address these questions, item-level network analysis offers a powerful framework. Network analysis is a statistical framework that conceptualizes psychopathology as a system of interacting symptoms rather than as manifestations of an underlying latent disorder [60,61,62]. In this approach, individual symptoms are represented as nodes, and the associations between them as edges, allowing for the identification of highly central symptoms that exert strong influence within the network, as well as bridge symptoms that connect distinct symptom clusters [63,64]. Network analysis has proved particular utility in clinical research because it can highlight potential mechanistic targets for intervention, reveal symptoms that maintain co-occurring psychopathology across domains, and distinguish network structures that are shared across disorders from those that are disorder-specific [65,66]. Moreover, computational modeling approaches highlight that the distinction between health and disorder is contingent upon the structural properties of the network [67]. From a network-oriented viewpoint, mental disorders are defined not only by the overall level of symptom activation within the system, but also by the underlying network architecture [64,68,69]. Specifically, when symptoms are tightly interconnected, their mutual interactions can sustain heightened activation states over time; in contrast, in networks with weaker interconnections, symptom activation tends to fluctuate along a continuum [70,71]. Highly connected networks, however, give rise to more abrupt transitions, as activation in any single component can quickly spread throughout the system, driving the network toward a pathological state. Applying a network framework to mental health enhances both conceptual understanding and clinical practice. By identifying the symptom-level pathways that sustain comorbidity and explain individual differences in diagnostic presentations, this approach clarifies how specific symptom interactions maintain disorders and, in turn, supports more precise and personalized interventions [72]. Importantly, to date, there are no network studies specifically examining combined samples of participants with AN, ANX, and MDD symptoms while analyzing measurement instruments at the item level. This gap highlights the pioneering nature of the present study and contextualizes its contribution, as it investigates transdiagnostic symptom interactions across these clinical populations using a detailed, item-level approach.

### Justification and Aims of the Study

The present study aims to characterize the symptom-level relationships among IU, body dissatisfaction, and eating attitudes across individuals with symptoms of AN, MDD, ANX and a control group (CG). Specifically, we aimed to (1) estimate item-level networks to identify central nodes within each domain, and (2) identify bridge nodes that link IU with body dissatisfaction and eating pathology. Given the pioneering and exploratory nature of this study, we did not formulate specific empirical hypotheses, as there is currently insufficient evidence to predict the precise network structure or the most relevant nodes.

The control group, CG, was specifically selected to include participants without symptoms of AN, ANX, or major depressive disorder, MDD. This allows for the differentiation of general, non-pathological associations between intolerance of uncertainty, eating-related attitudes, and body dissatisfaction from patterns that are specific to clinical conditions. By estimating networks within both clinical and control groups, we can compare the structure, strength, and relevance of nodes across populations, clarifying which mechanisms are transdiagnostic versus disorder-specific.

## 2. Materials and Methods

### 2.1. Participants and Procedure

Between 2019 and 2023, participants were recruited for a research program focused on transdiagnostic psychological processes related to MDD, ANX, and eating disorders across community samples, individuals with medical conditions, and people seeking psychological support. Recruitment took place through several channels, including online advertisements in peer-support Facebook groups, websites, and discussion forums, as well as newsletters circulated by relevant organizations and support services (e.g., MS Australia). Additional advertisements were displayed on noticeboards at the Royal Melbourne Hospital. Those interested in taking part were directed to complete an online survey. The study adhered to the Declaration of Helsinki and was approved by the University of Melbourne ethics committee (Project number: 2021-12495-20321-4; approval date: 21 March 2019). All participants gave written informed consent, confirming voluntary participation and the right to withdraw at any time.

All participants included in the present study were drawn from this research program and met the inclusion criteria established for that project (being aged 18 years or older and providing informed consent to participate in the online survey). No additional exclusion criteria were applied for the purposes of the current analyses. For this work, participants were subsequently classified into four groups based on their self-reported symptomatology: AN, MDD, ANX, or a control group. Group allocation was determined using participants’ responses to the self-report measures administered in the survey (see Section 2.2 for details). Individuals who did not meet criteria for AN, MDD, or ANX symptomatology were included in the control group. Table 1 displays the description of the sociodemographic characteristics of the participants in this study, as well as the total scores in the questionnaires employed in the work, stratified by group. The grouping labeled as “comorbid” includes individuals who reported the simultaneous presence of symptoms of at least two of the following conditions: AN, MDD, or ANX.

### 2.2. Measures

Participants completed an online survey comprising a set of standardized self-report questionnaires and basic demographic questions across two timepoints (baseline and at 6 months follow-up).

Eating Attitudes Test (EAT-26; [73]). This 26-item questionnaire was used to assess eating attitudes and behaviors, including dieting, bulimic behaviors, and preoccupation with food and weight. Items are rated on a 4-point Likert scale ranging from 0 (never) to 3 (always), with higher values reflecting greater eating disorder symptom severity. In the present sample, the EAT-26 achieved excellent internal consistency (Cronbach’s α = 0.923).

Body Shape Questionnaire (BSQ-8; [74]). This scale was employed to assess concerns related to body shape and weight, as well as distress associated with perceived body size. Participants are asked to indicate the frequency with which they experience body-related concerns over a specified period, using a Likert-type response format (higher scores indicate greater levels of body dissatisfaction). The internal consistency in this study was excellent (Cronbach’s α = 0.929).

Intolerance of Uncertainty Scale (IUS-27; [75]). This scale measures dispositional tendencies to perceive uncertain situations as stressful, upsetting, or threatening. Items assess emotional, cognitive, and behavioral reactions to uncertainty and ambiguity and are rated on a Likert scale, with higher total scores indicating greater IU. In the data file employed for this study, this tool obtained excellent internal consistency (Cronbach’s α = 0.958).

Demographic variables. Participants also provided basic demographic information, including sex, age, educational attainment, marital status, and self-identified ethnic group.

### 2.3. Statistical Analysis

Network analyses were conducted to examine the pattern of associations among eating disorder symptoms, body dissatisfaction, and IU. The network visualizations were performed using Gephi (version 0.9.2 for Windows), an open-source software specifically developed for the analysis of complex network structures [76]. Gephi allows both graphical representation and quantitative characterization of networks through the calculation of multiple topological indices. This software was chosen for the present study because it facilitates the clear visualization of complex interrelationships among nodes. Complementary analyses were conducted in R (version 4.5.3) using the packages qgraph and bootnet. Networks were estimated with the EBICglasso method (Extended Bayesian Information Criterion graphical least absolute shrinkage and selection operator). Additionally, stability analyses were carried out to evaluate the robustness of the estimated networks, and network comparison analyses were performed to identify differences between networks.

To evaluate the relative importance and functional role of individual nodes, three complementary centrality indices were calculated [77]: eigenvector centrality, betweenness centrality, and closeness centrality. Eigenvector centrality reflects the extent to which a node is connected to other highly connected nodes, thereby capturing its overall influence within the network. Betweenness centrality quantifies the frequency with which a node lies on the shortest paths linking pairs of nodes, providing an index of its potential role as an intermediary or connector between different regions of the network. Nodes with elevated betweenness are considered structurally critical, as their removal may substantially disrupt network cohesion. Closeness centrality represents the inverse of the average shortest path length between a given node and all other nodes, with higher values indicating greater proximity and more efficient access to the rest of the network.

Network modularity was estimated to identify the presence of substructures or communities within the network [78]. This procedure assesses the extent to which the network can be partitioned into modules characterized by dense internal connectivity and relatively sparse connections with other modules. The modularity algorithm implemented in Gephi enables data-driven detection of such clusters, facilitating the identification of meaningful groupings of variables without the need for predefined categories.

Several global network metrics were also computed to describe the overall architecture of the networks. Network density was used to quantify the proportion of observed edges relative to the total number of possible connections, serving as an index of general interconnectedness. The clustering coefficient was calculated to assess the tendency of nodes to form locally interconnected neighborhoods. Network efficiency was further characterized by the average path length, defined as the mean number of steps required to connect any two nodes via the shortest paths. In addition, network diameter was estimated as the maximum of these shortest paths, representing the greatest distance between any pair of nodes in the network.

Two separate networks were estimated for the clinical and control subsamples. The clinical group included all participants who presented symptoms associated with AN, ANX, or MDD simultaneously, as the purpose of the study was to contextualize the analyses from a transdiagnostic perspective. Although ideally networks would also be estimated separately for each subtype (AN, ANX, and MDD), this was not feasible because some subgroups were too small to reliably model networks as complex as those in the present study.

Nodes corresponded to individual item responses from the EAT-26, the BSQ-8, and the IUS-27, resulting in a total of 61 nodes per network.

## 3. Results

### 3.1. Results for the Clinical Subsample

**Figure 1:**
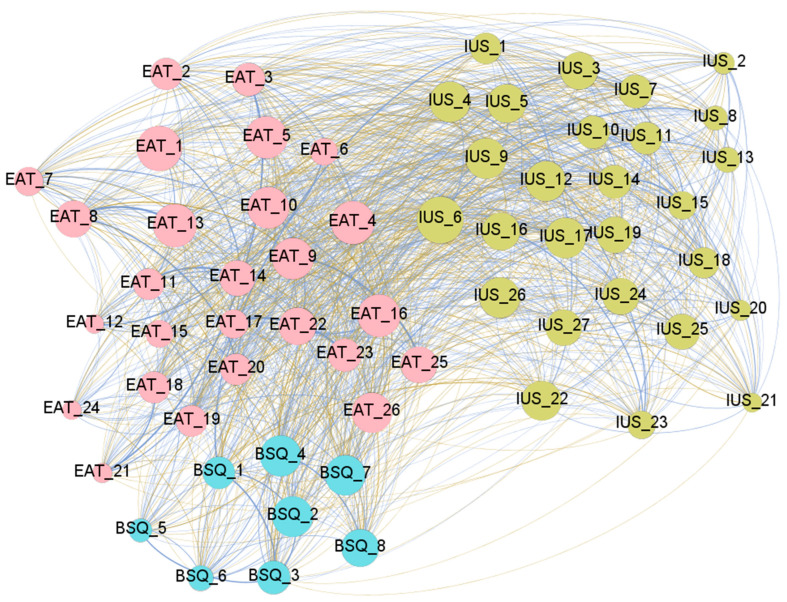
Visualization of the network obtained within the clinical subsample. Note. Blue edges: positive weights. Brown-ochre edges: negative weights. The thicker the edge, the stronger the edge. Nodes are plotted in colors depending on the dimension: eating attitudes (EAT-26 items, pink), body dissatisfaction (BSQ-8 items, blue), and intolerance to uncertainty (IUS-27 items, green). The higher the node, the higher the bridge’s expected impact. The correspondence between the full name and abbreviation of the nodes is shown in the Supplementary Materials.

Figure 1 presents the graph with the visualization of the network obtained within the clinical mental health sample. Table S1 (Supplementary Materials) contains the complete parameters obtained in the analysis. Regarding the network graph, the nodes are color-coded by dimension: eating attitudes (EAT-26 items, pink), body dissatisfaction (BSQ items, blue), and IU (IUS-27 items, green). Edges with positive weights are shown in blue to represent positive relationships (partial correlations *Rp* > 0), while negative weights are shown in ochre/brown to represent negative relationships (*Rp* < 0). The diameter of the network was 2, and the average path length was 1.251.

**Figure 2:**
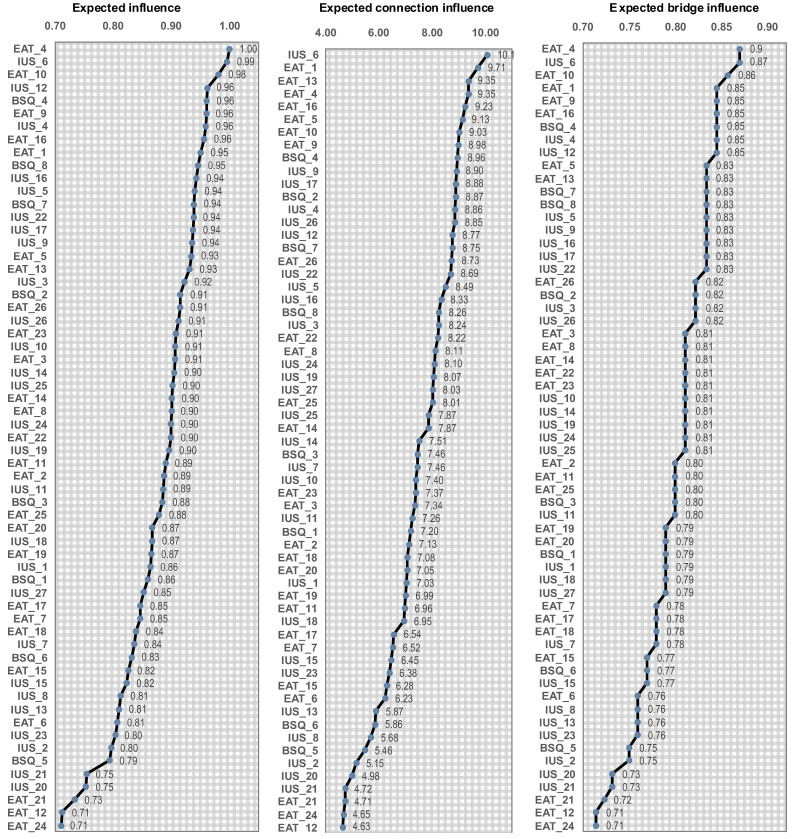
Expected influence, connection and influence of the nodes (clinical subsample). Note. Expected influence: eigenvector centrality. Expected connection: betweenness centrality. Expected bridge influence: closeness centrality. The correspondence between the full name and abbreviation of the nodes is shown in the Supplementary Materials.

Figure 2 displays the bar charts with the nodes ordered by their centrality coefficients. The item EAT_4 “I have gone on eating binges where I felt that I may not be able to stop” achieved the highest eigenvector and closeness, nearly followed by the node-items IUS_6 “uncertainty makes me uneasy, anxious, or stressed” and EAT_10 “I feel extremely guilty after eating”. The node-item IUS_6 also achieved the highest betweenness centrality, followed by node-item EAT_1, “I am terrified about being overweight”.

**Figure 3:**
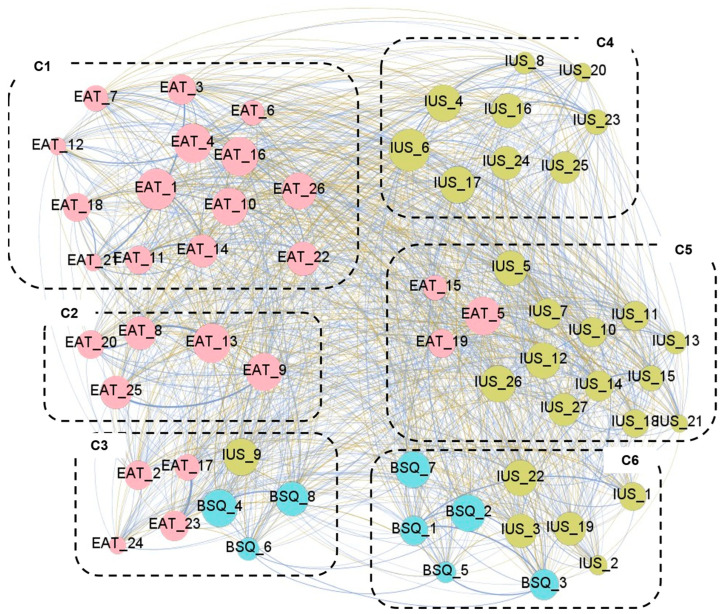
Visualization of the modularities in the network obtained within the clinical subsample. Note. Blue edges: positive weights. Brown-ochre edges: negative weights. The thicker the edge, the stronger the edge. Nodes are plotted in colors depending on the dimension: eating attitudes (EAT-26 items, pink), body dissatisfaction (BSQ-8 items, blue), and intolerance to uncertainty (IUS-27 items, green). The higher the node, the higher the bridge’s expected impact. C1 to C6: cluster 1 to cluster 6. The correspondence between the full name and abbreviation of the nodes is shown in the Supplementary Materials.

Six clusters of nodes were identified (modularity classes are inside the boxes of Figure 3). The modularity index, which describes how the network is classified into sub-networks and ranges from −1 to 1, was 0.265 (suggesting fair modularity), and the average clustering coefficient was 0.750. Clusters C1 and C2 group a large proportion of the EAT-16 items (15 and 5 items, respectively). C3 groups EAT-16 items referring to food restriction and dieting, together with item IUS_9 “uncertainty keeps me from living a full life” and three BSQ-8 items that assess avoidance of social situations due to discomfort related to body-related problems. The remaining IUS-27 items are distributed across clusters C4, C5 (together with three EAT-26 items that measure perceived self-control around food), and C6 (together with the remaining five BSQ-8 items).

Case-dropping bootstrap analyses (1000 samples) indicated that the network exhibited overall adequate stability, with strength (CS = 0.673) and closeness (CS = 0.517) emerging as the most reliable centrality measures, exceeding the recommended threshold of 0.50. Betweenness showed acceptable but lower stability (CS = 0.283). Strength remained highly stable across subsamples, while closeness declined only gradually. Betweenness declined more noticeably, reflecting lower stability. Edge-weight accuracy analyses (second plot, Figure S1, Supplementary Materials) showed high precision for the strongest connections, whereas weaker edges displayed greater variability and overlapping confidence intervals, a common pattern in bio-psychological networks.

### 3.2. Results for the Control Subsample

**Figure 4:**
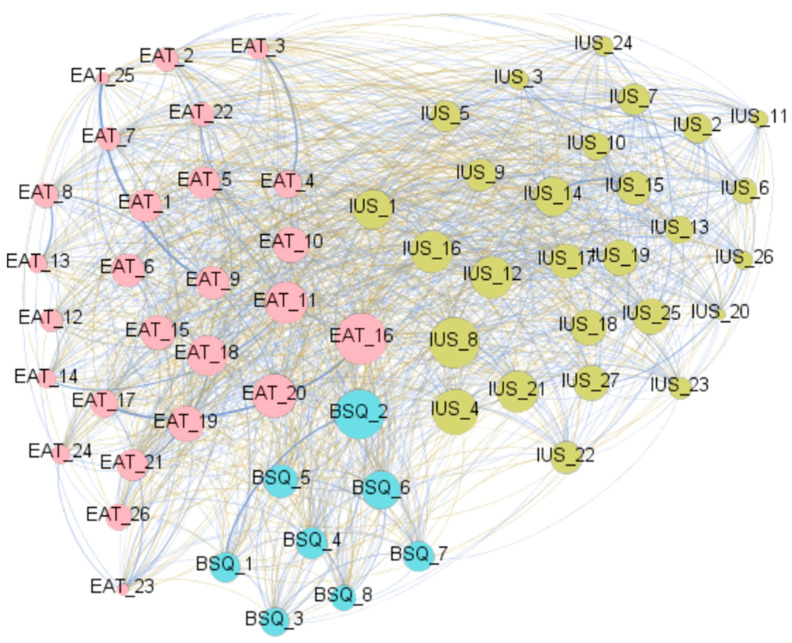
Visualization of the network obtained within the control subsample. Note. Blue edges: positive weights. Brown-ochre edges: negative weights. The thicker the edge, the stronger the edge. Nodes are plotted in colors depending on the dimension: eating attitudes (EAT-26 items, pink), body dissatisfaction (BSQ-8 items, blue), intolerance to uncertainty (IUS-27 items, green). The higher the node, the higher the bridge’s expected impact. The correspondence between the full name and abbreviation of the nodes is shown in the Supplementary Materials.

The graph with the visualization of the network for the CG is displayed in Figure 4 (Table S2, Supplementary Materials, contains the complete parameters obtained in the analysis). Nodes and edges are color-coded in the same way as the network for the clinical subsample. The diameter of the network was 2, and the average path length was 1.307.

**Figure 5:**
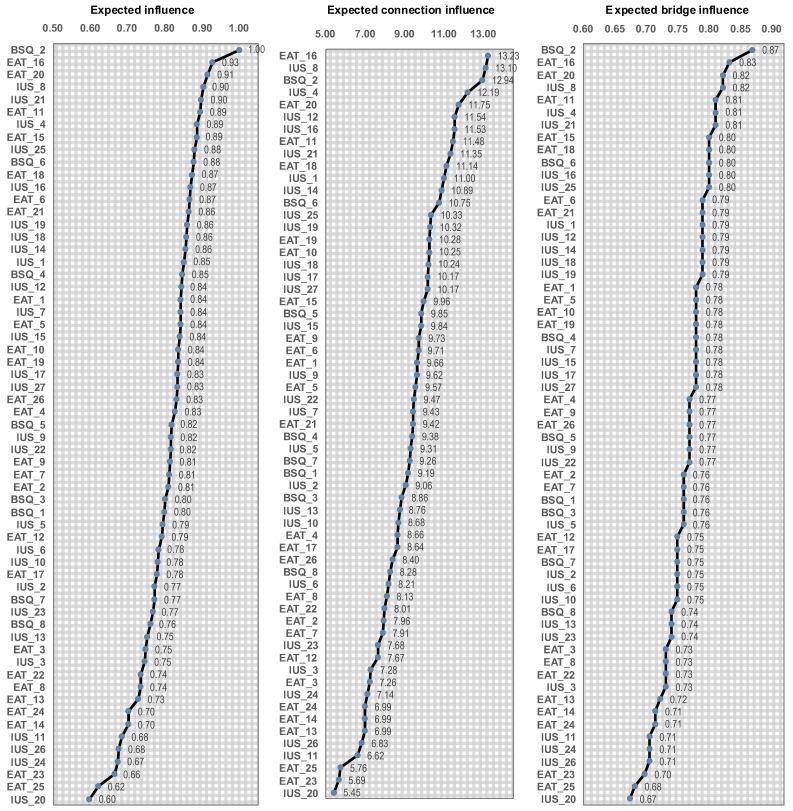
Expected influence, connection and influence of the nodes (control subsample). Note. Expected influence: eigenvector centrality. Expected connection: betweenness centrality. Expected bridge influence: closeness centrality. The correspondence between the full name and abbreviation of the nodes is shown in the Supplementary Materials.

The nodes ordered by their centrality are listed in Figure 5. The node-item BSQ_2 “felt my shape compared unfavorably to others” achieved the highest eigenvector centrality and closeness centrality. The highest betweenness centrality was achieved by EAT_16 “I avoid foods with sugar in them”, nearly followed by IUS_8 “it frustrates me not having all the information I need” and BSQ_2 “felt my shape compared unfavorably to others”.

**Figure 6:**
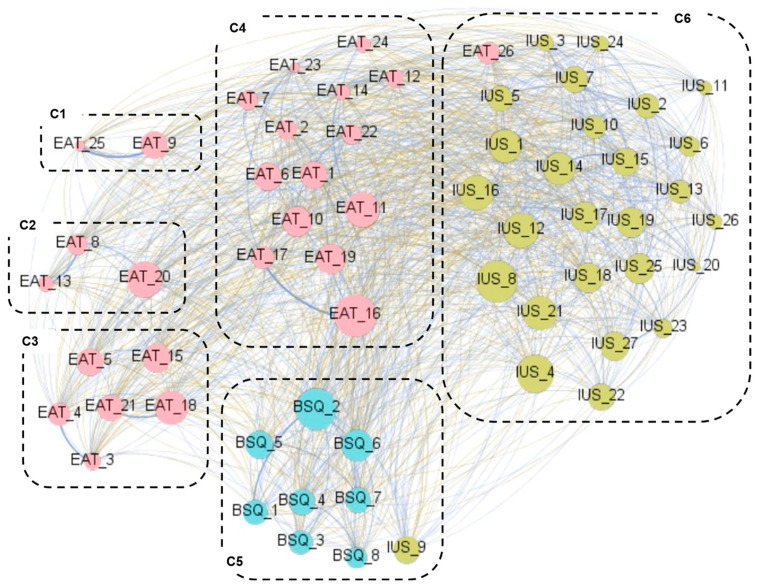
Visualization of the modularities in the network obtained within the control subsample. Note. Blue edges: positive weights. Brown-ochre edges: negative weights. The thicker the edge, the stronger the edge. Nodes are plotted in colors depending on the dimension: eating attitudes (EAT-26 items, pink), body dissatisfaction (BSQ-8 items, blue), and intolerance to uncertainty (IUS-27 items, green). The higher the node, the higher the bridge’s expected impact. C1 to C6: cluster 1 to cluster 6. The correspondence between the full name and abbreviation of the nodes is shown in the Supplementary Materials.

Six clusters of nodes were identified (modularity classes are inside the boxes of Figure 6), with a different composition compared with the grouping of the network for the clinical subsample. The modularity index was 0.368 (fair modularity), and the average clustering coefficient was 0.694. With regard to the composition of the clusters, the distribution of the node-items was as follows: (a) cluster C1 includes two EAT-26 items that measure the tendency to vomit after meals; (b) C2 includes EAT-26 items referring to others’ behavior in response to the own participant’s eating behavior; (c) C3 groups items from the EAT-16 that measure bulimia and food preocupations, as well as perceived self-control around food; (d) C4 groups a large proportion of the EAT-16 items included in the diet and control subscales; (e) C5 includes all BSQ-8 items, together with item IUS_9 “uncertainty keeps me from living a full live”; and (f) C6 includes the remaining IUS-27 items, together with item EAT_26 “I do not enjoy trying new rich foods”.

Bootstrap analyses (1000 samples) indicated good robustness for strength (CS = 0.672) and acceptable stability for betweenness (CS = 0.407) and closeness (CS = 0.517). Centrality stability (first plot, Figure S2, Supplementary Materials) showed high correlations with the original sample across all case-dropping levels, with strength and closeness particularly stable and betweenness slightly more variable but still acceptable. Edge-weight accuracy (second plot, Figure S2) revealed high precision for the strongest connections, while weaker edges exhibited greater variability and overlapping confidence intervals, a typical pattern in medical and mental health networks.

### 3.3. Comparison Between the Networks

The global network metrics for the clinical mental health and control samples revealed both similarities and differences. Both networks displayed an identical diameter (2.000), indicating that the overall connectivity and the longest shortest path within each network are comparable. However, differences were observed in the average path length, with the clinical sample showing a shorter path length (1.251) compared to the control sample (1.309), suggesting marginally more efficient information transfer in the clinical network. In terms of modularity, the CG network exhibited higher modularity (0.368) relative to the clinical network (0.265), indicating a more pronounced community structure among controls. Conversely, the average clustering coefficient was higher in the clinical network (0.750) than in the CG network (0.694), reflecting a tendency for stronger local clustering in the clinical subsample. Overall, these findings suggest differences in network organization, with the clinical network favoring local connectivity, whereas the CG network achieves greater community segregation.

The comparative analysis of the structural configuration of the networks between clinical patients and CG revealed clear differences. Nodes within the network in the clinical subsample corresponding to the EAT-26, IUS-27, and BSQ-8 groups cluster in a more fragmented manner, with specific interconnections within each cluster. In the network obtained among CG, the clusters exhibit a more compact and homogeneous organization, particularly for the BSQ-8 and the IUS-27 nodes. These variations in cluster arrangement indicate that the overall network structure differs between the two groups, providing evidence of alterations in the functional or relational organization of nodes in clinical patients compared to controls.

The analysis of node centrality measures (including eigenvector centrality, betweenness centrality, and bridge-related accessibility) provided converging evidence of differences in network organization between clinical patients and healthy controls. In the clinical network, nodes such as EAT_4 “I have gone on eating binges where I felt that I may not be able to stop”, the IUS_6 “uncertainty makes me uneasy, anxious, or stressed” consistently showed the highest eigenvector centrality and closeness centrality, and EAT_10 “I feel extremely guilty after eating”, indicating that these nodes are not only influential within the network but also function as effective bridges, maintaining short paths to a large proportion of other nodes. In addition, nodes with the highest betweenness centrality (IUS_6 “uncertainty makes me uneasy, anxious, or stressed” and EAT_1 “I am terrified about being overweight”) played a key role in connecting different regions of the network, suggesting that communication between subnetworks in the clinical group depends strongly on a limited set of connector nodes referred to as eating behavior and IU.

In contrast, the network within the control sample exhibited a distributed pattern across centrality indices. BSQ_2 registered the highest eigenvector centrality and closeness centrality, while closeness centrality was spread across a broader range of nodes: EAT_16 “I avoid foods with sugar in them”, IUS_8 “it frustrates me not having all the information I need”, and BSQ_2 “felt my shape compared unfavorably to others”. This pattern indicates that relevance and bridge-like access to the network are mainly concentrated in a specific node, but connectivity between different network regions is less concentrated, reflecting a more integrated and evenly connected structure.

The statistical comparison between networks revealed notable differences in several centrality measures. Degree centrality exhibited a moderate and statistically significant correlation (r = 0.48, 95% CI [0.26, 0.66], *p* < 0.001), suggesting a partial overlap among the most directly connected nodes across networks. In contrast, betweenness (r = 0.09, 95% CI [−0.17, 0.33], *p* = 0.488) and closeness (r = 0.21, 95% CI [−0.05, 0.44], *p* = 0.112) were not significantly correlated, indicating that nodes functioning as bridges between communities and those occupying central positions in terms of shortest paths differ substantially between networks. These results highlight that while some aspects of network connectivity are shared, the structural roles of nodes beyond direct connections can vary considerably, reflecting nuanced differences in network organization.

## 4. Discussion

The present study aimed to examine the network structure of eating attitudes, body dissatisfaction, and IU in a sample of patients who reported symptoms for AN, MDD, and ANX, compared with a CG. Specifically, we investigated differences in global network organization, community structure, and node centrality to elucidate the relational architecture of symptoms across the two samples. The results revealed clear differences between the two networks: the clinical mental health network was characterized by a shorter average path length and higher clustering, whereas the control network showed higher modularity and a more segregated yet integrated structure. Moreover, centrality patterns differed substantially between groups, suggesting distinct mechanisms underlying symptom interrelations.

It is important to note that this study is cross-sectional and exploratory in nature, which limits causal inference and warrants caution when interpreting clinical or nutritional implications.

### 4.1. Network in the Clinical Subsample

In the clinical mental health sample, the network displayed a highly clustered structure, characterized by several nodes showing high levels of relevance, as indicated by elevated eigenvector centrality, and occupying key positions in terms of closeness centrality across symptom domains related to eating pathology and IU. From a network psychopathology perspective, this pattern of centrality suggests the presence of self-reinforcing symptom processes, whereby highly interconnected nodes related to eating behavior and IU exert a disproportionate influence on the overall dynamics of the network. Specifically, items tapping binge eating, guilt following eating, fear of weight gain, and negative moods related to uncertainty emerged as the most central components, suggesting that eating-related behaviors and uncertainty-related distress may act as core drivers of symptom activation and maintenance. Notably, no items from the BSQ-8 emerged among the most relevant or highly connected nodes in this network, indicating that concerns related to body shape may play a less central role in the clinical symptom structure when compared to eating behaviors and uncertainty-related processes. This finding suggests that, in the clinical mental health sample, body dissatisfaction may be more peripheral or downstream, rather than functioning as a primary organizing feature of the network [79].

This pattern aligns with transdiagnostic models that conceptualize IU as a higher-order vulnerability factor that amplifies negative affect and fosters maladaptive coping strategies across mental disorders [80]. Results are also consistent with studies that observed that trait and disorder-specific IU are significantly associated with multiple cognitive vulnerability factors, with direct and indirect effects on the presence of multiple mental symptoms [81]. In patients with AN, elevated IU and fear of weight gain have been identified as central mechanisms contributing to the persistence of restrictive and binge–purge behaviors [27], which are often further reinforced by guilt and self-critical cognitions following eating episodes [82,83]. In ANX, high levels of IU have been consistently identified as a core mechanism shaping excessive worry and behavioral avoidance [84], which can generalize to the adoption and maintenance of rigid behavioral patterns across anxiety presentations [6]. Finally, depressive symptomatology has often been conceptualized as involving a cognitive stance characterized by perceived inevitability rather than uncertainty. Within this framework, theoretical accounts such as the Hopelessness Theory of Depression [85] describe a subtype of depression in which entrenched negative expectations (reflecting a conviction-certainty that future circumstances are unlikely to improve) play a central etiological role. Depressive manifestations have likewise been linked to a cognitive orientation marked by increased confidence in the occurrence of adverse future events and the improbability of positive outcomes [86]. At the same time, accumulating empirical evidence indicates substantial phenomenological overlap between anxiety and depressive presentations, supporting conceptualizations of IU as a transdiagnostic process operating across both conditions when they are viewed dimensionally rather than as discrete diagnostic categories [87].

Importantly, the fragmented clustering observed in the clinical network suggests that these disorder-specific processes do not operate independently, but rather converge through a restricted set of highly influential connector nodes. From a dynamic network perspective, such an architecture implies that communication between symptom communities relies heavily on bridge symptoms, lowering the threshold for cross-domain activation [65,88]. As a consequence, activation within one domain (such as uncertainty-related distress or eating-related guilt) may rapidly propagate to other symptom clusters through these connectors, producing cascading effects across the network. This configuration is consistent with dynamic models of symptom maintenance and relapse [64], which emphasize the role of critical nodes and feedback loops in driving sudden symptom escalation and reducing system resilience. Accordingly, the tightly interconnected yet fragmented structure of the clinical mental health network may contribute to symptom persistence, heightened comorbidity, and increased vulnerability to relapse in individuals with co-occurring ANX, MDD, and AN.

### 4.2. Network in the Control Subsample

In the CG, the nodes that emerged as particularly central provide relevant insight into cognitive and behavioral processes that may confer vulnerability to clinical symptomatology. Specifically, BSQ_2 (“felt my shape compared unfavorably to others”) showed the highest eigenvector and closeness centrality, suggesting that body dissatisfaction occupies a pivotal position even among non-clinical individuals. Elevated levels of body dissatisfaction have consistently been associated with increased vulnerability to AN [89], as negative self-evaluations related to body shape and weight may foster restrictive eating patterns and persistent preoccupations with food and dietary control. This finding also suggests that body dissatisfaction may constitute a shared psychological vulnerability among individuals without AN [90]. Recent longitudinal research has shown that higher body dissatisfaction in adolescence predicts later eating disorder symptomatology in general population cohorts [91] and that body dissatisfaction and disordered eating exhibit prospective associations over time [92]. Additionally, survey data from young adult samples indicate robust associations between body dissatisfaction and maladaptive eating behaviors in non-clinical university populations [90]. The cumulative evidence suggests that the pervasive influence of body dissatisfaction on cognitive, emotional, behavioral, and social domains highlights its role as a transdiagnostic factor with significant implications for emotional well-being, functional impairment, and overall health.

The items EAT_16 (“I avoid foods with sugar in them”) and IUS_8 (“it frustrates me not having all the information I need”) exhibited the highest betweenness centrality, indicating that these nodes may function as bridges linking cognitive vulnerabilities and behavioral manifestations. Dietary restraint behaviors, such as the avoidance of sugar-containing foods, have been widely documented as early markers of disordered eating trajectories and may precede or reinforce symptom patterns characteristic of AN [93,94]. Moreover, restrictive dietary practices have also been associated with elevated depressive symptomatology: large observational data indicate that adherence to calorie- or nutrient-restrictive diets is linked to higher depressive symptom scores in community samples [95], and broader dietary patterns low in variety or rich in processed foods are correlated with increased depression risk [96,97], potentially through mechanisms involving excessive self-regulation, guilt related to food consumption, and reduced dietary flexibility. Additionally, research on emotion dysregulation suggests that rigid eating rules and self-criticism related to food consumption may reinforce maladaptive self-regulation mechanisms that overlap with depressive processes [98].

On the other hand, IU is a well-established cognitive vulnerability in ANX, consistently implicated in excessive worry and related internalizing symptoms across clinical and non-clinical samples (e.g., intolerance of uncertainty contributes to the maintenance of anxiety and worry processes in generalized anxiety and other internalizing conditions) [7]. Moreover, IU has been linked empirically to cognitive rigidity and avoidance behaviors, and research in eating disorder samples indicates that higher IU is associated with restrictive eating patterns, worry about dietary ambiguity, and a rigid cognitive style [26]. Emerging evidence further suggests that individuals with elevated IU may rely increasingly on rigid eating rules and detailed nutritional information in an attempt to reduce perceived uncertainty and ambiguity surrounding food choice, which in turn may fuel inflexible dietary behaviors [33].

Taken together, the centrality of body dissatisfaction, dietary restraint, and intolerance of uncertainty suggests that, even within a non-clinical population, these processes are closely interconnected. Previous evidence indicates that their co-occurrence may be associated with maladaptive eating patterns and cognitive rigidity linked with anxiety and restrictive eating behaviors, consistent with transdiagnostic models of intolerance of uncertainty that connect worry, avoidance, and cognitive inflexibility with eating pathology in non-clinical samples. Furthermore, dietary restriction and patterns low in variety have been linked with poorer mental health outcomes, including elevated depressive symptomatology and psychological distress, which could represent early risk configurations for the later development or intensification of AN, MDD, or ANX symptomatology. Nutritional research likewise highlights that lack of dietary variety and restrictive patterns may increase vulnerability to negative mood and affective symptoms, potentially via neurobiological and psychosocial mechanisms related to diet quality and stress responses.

Regarding the clusters of nodes, the network structure observed in the CG was more modular and evenly organized than the clinical network, with groups of nodes reflecting body dissatisfaction, eating attitudes, and IU maintaining relative independence. Although body dissatisfaction nodes exhibited relatively high centrality, overall influence was distributed across multiple eating- and uncertainty-related nodes, resulting in a more balanced configuration in which no single symptom dominated information flow. From a network psychopathology perspective, such a distributed structure is theoretically associated with greater system stability, as the activation of any single node is less likely to trigger cascading effects across other symptom domains [64,65,68,99]. This modular and relatively homogeneous organization is also consistent with models of resilience conceptualized as a dynamic and distributed process rather than the product of a single protective factor [100]. Moreover, it aligns with models of normative cognitive–emotional functioning, in which negative affect and IU operate as transdiagnostic but context-sensitive processes that do not necessarily escalate into maladaptive behavioral patterns [80,101,102]. In this context, body dissatisfaction and uncertainty-related experiences may function as normative stressors that inform behavior without overwhelming the broader system, thereby supporting flexible coping strategies and adaptive regulation of emotions and eating behaviors [79,103]. Compared to the clinical mental health network, the control network appears more resistant to destabilization, with connectivity and centrality more evenly distributed across nodes, reducing the likelihood of symptom amplification and promoting psychological resilience in the face of everyday stressors.

### 4.3. Limitations and Strengths

Several limitations should be acknowledged. First, the cross-sectional design precludes causal inferences regarding the directionality of relationships between nodes. Second, although the use of item-level networks allows for fine-grained analysis, it also increases model complexity and may limit generalizability. Future research should incorporate longitudinal designs to examine network dynamics over time and assess whether changes in central nodes predict clinical improvement or relapse. Third, participants with symptoms related to AN, MDD, or ANX were combined into a single pooled sample to estimate a common network, in line with a transdiagnostic perspective. Estimating separate, diagnosis-specific networks was not feasible due to limited sample sizes within each subgroup, which means that the resulting network may represent a combination of distinct mechanisms rather than a purely shared transdiagnostic structure. Moreover, differences in the phenotypes associated with each clinical condition, including sociodemographic characteristics, could contribute to some of the connections identified in the network. Future studies with larger and more balanced samples should assess networks separately by diagnostic category and formally compare them, which would allow the investigation of potential heterogeneity across subtypes and provide a more nuanced understanding of how symptom domains interact across different clinical profiles. A limitation of this study was also that the classification of participants into AN, ANX, and MDD symptom groups was based on self-report rather than clinical verification. Moreover, the sample comprises individuals with symptoms associated with these conditions, rather than participants meeting full clinical diagnostic criteria. Nevertheless, the instruments used are well-validated and have strong empirical support for their reliability. Finally, another limitation concerns the absence of detailed inclusion and exclusion criteria related to the clinical characteristics of the condition, including the current phase of the disorder (e.g., acute, remission), clinical course, severity, and current or past treatment. This limitation may have contributed to increased heterogeneity within the sample, which could partially account for variability in the findings and represent a significant source of bias with respect to the study aims. Future research would benefit from incorporating these factors to enhance the specificity and interpretability of the results.

Despite these limitations, the study also presents notable strengths. These include the integration of multiple theoretically relevant domains and the use of both global and local network metrics to characterize symptom organization. By moving beyond mean-level comparisons, the present findings contribute to a more nuanced understanding of how symptoms are interconnected in clinical mental health versus non-clinical populations.

### 4.4. Implications

In the clinical mental health network, highly central and bridging nodes (such as binge eating behaviors, eating-related guilt, and IU) represent key targets for intervention. Network-informed strategies that focus on these symptoms could disrupt maladaptive connectivity patterns, reduce the spread of disordered behaviors, and enhance treatment efficacy. Integrating these approaches into structured cognitive-behavioral and nutrition-focused programs may allow clinicians to directly address the most influential processes sustaining eating pathology, including maladaptive dietary habits and affective responses to food.

The following clinical and practical implications are based on the overall patterns observed in the study; however, they should not be interpreted as directly testable or derivable from the specific empirical results, given the exploratory and cross-sectional design.

A nutrition-focused program could involve individualized meal planning to reduce the likelihood of binge episodes, education on balanced macronutrient intake, and strategies to manage food-related guilt. Practical interventions may include guided exposure to previously avoided foods, mindful eating exercises to enhance awareness of hunger and satiety cues, and structured problem-solving to manage uncertainty around food choices. By combining cognitive-behavioral techniques with these nutrition-specific strategies, clinicians can target both the emotional and behavioral drivers of disordered eating, helping patients to develop adaptive eating patterns and reduce reliance on maladaptive coping mechanisms.

In non-clinical populations, the more distributed network organization highlights the role of resilience mechanisms that prevent symptom consolidation. Nodes such as body dissatisfaction, dietary restraint, and sensitivity to uncertainty, while non-pathological, may serve as early indicators of vulnerability. Preventive strategies could include structured nutrition-focused programs that teach balanced eating habits, encourage exposure to a variety of foods, and reduce rigid dietary restraint, directly addressing tendencies reflected in EAT_16 “I avoid foods with sugar in them”. Psychoeducation on healthy body image and self-perception could target cognitive patterns linked to BSQ_2 “felt my shape compared unfavorably to others”, helping individuals critically evaluate negative comparisons and develop adaptive self-evaluation strategies.

Additionally, training in uncertainty and stress management could mitigate the impact of IUS_8, “it frustrates me not having all the information I need”, providing coping skills to handle food-related or daily life decisions without escalating worry or anxiety. Programs may combine interactive workshops, guided meal planning exercises, and mindfulness-based eating interventions to promote awareness of hunger and satiety cues, reduce food-related guilt, and reinforce adaptive coping strategies. Monitoring these processes over time can help identify individuals at elevated risk for clinically significant eating, depressive, or anxiety symptoms, allowing for early, tailored interventions that strengthen resilience and prevent the consolidation of maladaptive symptom networks.

## Figures and Tables

**Table 1:** Description for the sample.

		CG		AN		MDD		ANX		Comorbid	
		*N* = 842		*N* = 105		*N* = 97		*N* = 240		*N* = 84	
		*n*	%	*n*	%	*n*	%	*n*	%	*n*	%
Gender	Female	596	70.8%	68	64.8%	87	89.7%	216	90.0%	77	91.7%
	Male	246	29.2%	37	35.2%	10	10.3%	24	10.0%	7	8.3%
Marital	Single	726	86.2%	27	25.7%	50	51.5%	111	46.3%	42	50.0%
	Married	19	2.3%	73	69.5%	20	20.6%	71	29.6%	20	23.8%
	Divorced-widowed	97	11.5%	5	4.8%	27	27.8%	58	24.2%	22	26.2%
Ethnicity	African American	7	0.8%	8	7.6%	1	1.0%	7	2.9%	5	6.0%
	Caucasian	192	22.8%	63	60.0%	72	74.2%	178	74.2%	59	70.2%
	Eastern Asian	365	43.3%	3	2.9%	6	6.2%	14	5.8%	7	8.3%
	Southern Asian	139	16.5%	3	2.9%	4	4.1%	13	5.4%	1	1.2%
	Hispanic	3	0.4%	2	1.9%	2	2.1%	4	1.7%	2	2.4%
	Middle Eastern	19	2.3%	6	5.7%	1	1.0%	2	0.8%	2	2.4%
	Native Hawaiian	1	0.1%	1	1.0%	0	0.0%	0	0.0%	0	0.0%
	Other	114	13.5%	2	1.9%	8	8.2%	19	7.9%	6	7.1%
	Indigenous Austr.	2	0.2%	17	16.2%	3	3.1%	3	1.3%	2	2.4%
Origin	Australia	585	69.5%	74	70.5%	70	72.2%	199	82.9%	52	61.9%
	Other	257	30.5%	31	29.5%	27	27.8%	41	17.1%	32	38.1%
Employm.	Employed	424	50.4%	102	97.1%	59	60.8%	181	75.4%	65	77.4%
	Unemployed	418	49.6%	3	2.9%	38	39.2%	59	24.6%	19	22.6%
		Mean	SD	Mean	SD	Mean	SD	Mean	SD	Mean	SD
Age	Years-old	19.83	3.70	30.71	8.33	28.61	11.07	29.78	12.16	27.21	9.54
Eatting	EAT-26 total	11.73	12.28	25.65	14.50	15.42	15.32	15.31	12.64	25.57	17.37
Body	BSQ total	22.61	10.20	28.00	7.32	27.70	11.77	26.40	9.66	32.73	9.52
Uncertainty	IUS total	67.71	21.62	82.20	14.70	77.29	22.89	75.33	17.77	81.70	21.85

Note. CG: control group. AN: anorexia nervosa symptoms. MDD: major depressive disorder symptoms. ANX: anxiety disorder symptoms. Comorbid: comorbid condition. SD: standard deviation.

## Data Availability

Restrictions on data availability due to ethical considerations. The raw data supporting the conclusions of this article will be made available by the authors, provided that the request complies with the ethical regulations of the institution.

## Supplementary Materials

The following supporting information can be downloaded at: https://www.mdpi.com/article/10.3390/nu18091370/s1, Table S1: Results of the network within the clinical subsample. Table S2: Results of the network within the control subsample. Figure S1: Centrality analysis, stability plot and edge-weight accuracy plot within the clinical subsample. Figure S2: Centrality analysis, stability plot and edge-weight accuracy plot within the control subsample.
