# Supplementary material for: Uncertainty Meets Disordered Eating and Body Image: A Transdiagnostic Network Study Across Depressive, Anxiety and Anorexia Nervosa Symptoms Including a Control Group
Source: Nutrients. 2026 Apr 27;18(9):1370. doi: 10.3390/nu18091370 (PMC13165212; doi:10.3390/nu18091370)

SUPPLEMENTARY MATERIAL

Table S1 *Results of the network within the clinical subsample*

Label	Dimension	ID	Closeness centrality	Harmonic closeness centrality	Betweeness centrality	Authority	Modularity	Clustering. coefficient	Number triangles	Eigenvector centrality
EAT_1	Eating	Terrified about being overweight	0.8451	0.9083	9.7138	0.1378	1	0.7347	864	0.9502
EAT_2	Eating	Avoid eating	0.8000	0.8750	7.1272	0.1287	3	0.7586	751	0.8862
EAT_3	Eating	Preoccupied with food	0.8108	0.8833	7.3395	0.1312	1	0.7614	788	0.9062
EAT_4	Eating	Eating binges	0.8696	0.9250	9.3485	0.1585	1	0.7553	963	1.0000
EAT_5	Eating	Cut my food into small pieces	0.8333	0.9000	9.1253	0.1384	6	0.7376	832	0.9336
EAT_6	Eating	Aware of the calories	0.7595	0.8417	6.2273	0.1165	1	0.7488	614	0.8065
EAT_7	Eating	Avoid carbohydrates	0.7792	0.8583	6.5213	0.1225	1	0.7586	685	0.8456
EAT_8	Eating	Others prefer if I ate more	0.8108	0.8833	8.1123	0.1303	2	0.7430	769	0.8994
EAT_9	Eating	Vomits	0.8451	0.9083	8.9835	0.1390	2	0.7474	879	0.9595
EAT_10	Eating	Guilty after eating	0.8571	0.9167	9.0264	0.1422	1	0.7543	924	0.9803
EAT_11	Eating	Eesire to be thinner	0.8000	0.8750	6.9641	0.1286	1	0.7616	754	0.8892
EAT_12	Eating	Exercise	0.7143	0.8000	4.6252	0.1004	1	0.7540	475	0.7110
EAT_13	Eating	Others think I am too thin	0.8333	0.9000	9.3495	0.1349	2	0.7323	826	0.9313
EAT_14	Eating	Preoccupied of fat on my body	0.8108	0.8833	7.8702	0.1304	1	0.7498	776	0.9004
EAT_15	Eating	Take long to eat meals	0.7692	0.8500	6.2790	0.1193	6	0.7561	651	0.8247
EAT_16	Eating	Avoid sugar	0.8451	0.9083	9.2264	0.1384	1	0.7440	875	0.9554
EAT_17	Eating	Eat diet foods	0.7792	0.8583	6.5421	0.1230	3	0.7619	688	0.8457
EAT_18	Eating	Food controls my life	0.7792	0.8583	7.0766	0.1213	1	0.7453	673	0.8382
EAT_19	Eating	Self-control around food	0.7895	0.8667	6.9872	0.1253	6	0.7537	713	0.8654
EAT_20	Eating	Others pressure me to eat	0.7895	0.8667	7.0477	0.1256	2	0.7516	711	0.8667
EAT_21	Eating	Time / thoughts to food	0.7229	0.8083	4.7104	0.1061	1	0.7568	504	0.7329
EAT_22	Eating	Uncomfortable eating sweets	0.8108	0.8833	8.2164	0.1302	1	0.7401	766	0.8984

Label	Dimension	ID	Closeness centrality	Harmonic closeness centrality	Betweeness centrality	Authority	Modularity	Clustering. coefficient	Number triangles	Eigenvector centrality
EAT_23	Eating	Dieting behaviour	0.8108	0.8833	7.3709	0.1315	3	0.7585	785	0.9077
EAT_24	Eating	Like my stomach to be empty	0.7143	0.8000	4.6508	0.1027	3	0.7492	472	0.7091
EAT_25	Eating	Impulse to vomit after meals	0.8000	0.8750	8.0121	0.1272	2	0.7384	731	0.8776
EAT_26	Eating	Enjoy trying new rich foods	0.8219	0.8917	8.7311	0.1325	1	0.7401	800	0.9144
BSQ_1	Body shape	Worried about shape	0.7895	0.8667	7.2024	0.1242	4	0.7505	710	0.8591
BSQ_2	Body shape	Compare body shape	0.8219	0.8917	8.8747	0.1325	4	0.7345	794	0.9144
BSQ_3	Body shape	Felt fat when naked	0.8000	0.8750	7.4607	0.1255	4	0.7495	742	0.8844
BSQ_4	Body shape	Avoid social situations	0.8451	0.9083	8.9613	0.1392	3	0.7474	879	0.9607
BSQ_5	Body shape	Worried others seeing fat	0.7500	0.8333	5.4567	0.1152	4	0.7615	594	0.7938
BSQ_6	Body shape	Worried taking up much space	0.7692	0.8500	5.8592	0.1209	3	0.7689	662	0.8319
BSQ_7	Body shape	Check fat levels	0.8333	0.9000	8.7488	0.1356	4	0.7456	841	0.9378
BSQ_8	Body shape	Avoid situations	0.8333	0.9000	8.2584	0.1345	3	0.7571	854	0.9458
IUS_1	Uncertainty	Stops having a firm opinion	0.7895	0.8667	7.0325	0.1253	4	0.7505	710	0.8637
IUS_2	Uncertainty	Disorganized	0.7500	0.8333	5.1453	0.1153	4	0.7718	602	0.7955
IUS_3	Uncertainty	Intolerable	0.8219	0.8917	8.2425	0.1337	4	0.7465	807	0.9222
IUS_4	Uncertainty	Unfair not having guarantees	0.8451	0.9083	8.8598	0.1206	5	0.7500	882	0.9592
IUS_5	Uncertainty	Mind unrelaxed	0.8333	0.9000	8.4932	0.1391	6	0.7509	847	0.9392
IUS_6	Uncertainty	Uneasy, anxious, or stressed	0.8696	0.9250	10.0765	0.1442	5	0.7412	945	0.9948
IUS_7	Uncertainty	Unforeseen events upset	0.7792	0.8583	7.4566	0.1185	6	0.7331	662	0.8360
IUS_8	Uncertainty	Frustation for not having information	0.7595	0.8417	5.6803	0.1209	5	0.7622	625	0.8121
IUS_9	Uncertainty	Keeps me from living a full life	0.8333	0.9000	8.9009	0.1355	3	0.7438	839	0.9357
IUS_10	Uncertainty	Avoid surprises	0.8108	0.8833	7.3969	0.1319	6	0.7604	787	0.9064
IUS_11	Uncertainty	Unforeseen event can spoil	0.8000	0.8750	7.2555	0.1287	6	0.7556	748	0.8850
IUS_12	Uncertainty	Uncertainty paralyses me	0.8451	0.9083	8.7700	0.1397	6	0.7517	884	0.9610
IUS_13	Uncertainty	I am not first rate	0.7595	0.8417	5.8710	0.1177	6	0.7549	619	0.8099

Label	Dimension	ID	Closeness centrality	Harmonic closeness centrality	Betweeness centrality	Authority	Modularity	Clustering. coefficient	Number triangles	Eigenvector centrality
IUS_14	Uncertainty	I can't go forward	0.8108	0.8833	7.5140	0.1310	6	0.7556	782	0.9040
IUS_15	Uncertainty	I can't function very well	0.7692	0.8500	6.4486	0.1193	6	0.7515	647	0.8223
IUS_16	Uncertainty	Others know where they are going	0.8333	0.9000	8.3275	0.1365	5	0.7544	851	0.9427
IUS_17	Uncertainty	Vulnerable, unhappy, or sad	0.8333	0.9000	8.8827	0.1359	5	0.7429	838	0.9366
IUS_18	Uncertainty	I want to know what the future	0.7895	0.8667	6.9483	0.1255	6	0.7537	713	0.8664
IUS_19	Uncertainty	I can't stand being taken by surprise	0.8108	0.8833	8.0682	0.1330	4	0.7440	770	0.8959
IUS_20	Uncertainty	Smallest doubt can stop me	0.7317	0.8167	4.9839	0.1087	5	0.7596	534	0.7524
IUS_21	Uncertainty	Organize everything in advance	0.7317	0.8167	4.7150	0.1096	6	0.7681	540	0.7530
IUS_22	Uncertainty	I lack confidence	0.8333	0.9000	8.6878	0.1332	4	0.7473	843	0.9375
IUS_23	Uncertainty	Unfair other seem sure about future	0.7595	0.8417	6.3806	0.1161	5	0.7439	610	0.8027
IUS_24	Uncertainty	Don't sleep soundly	0.8108	0.8833	8.0993	0.1302	5	0.7449	771	0.8988
IUS_25	Uncertainty	Get away from uncertain situations	0.8108	0.8833	7.8713	0.1310	5	0.7498	776	0.9012
IUS_26	Uncertainty	Ambiguities stress me	0.8219	0.8917	8.8538	0.1319	6	0.7354	795	0.9117
IUS_27	Uncertainty	I can't stand being undecided	0.7895	0.8667	8.0311	0.1209	6	0.7262	687	0.8520

Table S2 *Results of the network within the control subsample*

Label	Dimension	ID	Closeness centrality	Harmonic closeness centrality	Betweeness centrality	Authority	Modularity	Clustering. coefficient	Number triangles	Eigenvector centrality
EAT_1	Eating	Terrified about being overweight	0.7792	0.8583	9.6588	0.1331	2	0.6988	631	0.8435
EAT_2	Eating	Avoid eating	0.7595	0.8417	7.9642	0.1278	2	0.7183	589	0.8098
EAT_3	Eating	Preoccupied with food	0.7317	0.8167	7.2603	0.1182	3	0.7055	496	0.7493
EAT_4	Eating	Eating binges	0.7692	0.8500	8.6605	0.1307	3	0.7143	615	0.8283
EAT_5	Eating	Cut my food into small pieces	0.7792	0.8583	9.5742	0.1329	3	0.7032	635	0.8425
EAT_6	Eating	Aware of the calories	0.7895	0.8667	9.7073	0.1367	2	0.7104	672	0.8663
EAT_7	Eating	Avoid carbohydrates	0.7595	0.8417	7.9084	0.1282	2	0.7207	591	0.8121
EAT_8	Eating	Others prefer if I ate more	0.7317	0.8167	8.1307	0.1159	1	0.6814	479	0.7350
EAT_9	Eating	Vomits	0.7692	0.8500	9.7305	0.1285	4	0.6934	597	0.8148
EAT_10	Eating	Guilty after eating	0.7792	0.8583	10.2535	0.1320	2	0.6844	618	0.8368
EAT_11	Eating	Eesire to be thinner	0.8108	0.8833	11.4752	0.1411	2	0.6928	717	0.8946
EAT_12	Eating	Exercise	0.7500	0.8333	7.6698	0.1251	2	0.7167	559	0.7926
EAT_13	Eating	Others think I am too thin	0.7229	0.8083	6.9869	0.1151	1	0.7027	468	0.7295
EAT_14	Eating	Preoccupied of fat on my body	0.7143	0.8000	6.9882	0.1108	2	0.6905	435	0.7024
EAT_15	Eating	Take long to eat meals	0.8000	0.8750	9.9645	0.1397	3	0.7121	705	0.8854
EAT_16	Eating	Avoid sugar	0.8333	0.9000	13.2349	0.1464	2	0.6817	769	0.9279
EAT_17	Eating	Eat diet foods	0.7500	0.8333	8.6417	0.1229	2	0.6949	542	0.7791
EAT_18	Eating	Food controls my life	0.8000	0.8750	11.1397	0.1377	3	0.6899	683	0.8728
EAT_19	Eating	Self-control around food	0.7792	0.8583	10.2823	0.1319	2	0.6888	622	0.8358
EAT_20	Eating	Others pressure me to eat	0.8219	0.8917	11.7495	0.1443	1	0.6957	752	0.9145
EAT_21	Eating	Time / thoughts to food	0.7895	0.8667	9.4248	0.1363	3	0.7178	679	0.8641
EAT_22	Eating	Uncomfortable eating sweets	0.7317	0.8167	8.0077	0.1161	2	0.6885	484	0.7361
EAT_23	Eating	Dieting behaviour	0.6977	0.7833	5.6852	0.1048	2	0.7130	400	0.6642
EAT_24	Eating	Like my stomach to be empty	0.7143	0.8000	6.9894	0.1109	2	0.6984	440	0.7029

Label	Dimension	ID	Closeness centrality	Harmonic closeness centrality	Betweeness centrality	Authority	Modularity	Clustering. coefficient	Number triangles	Eigenvector centrality
EAT_25	Eating	Impulse to vomit after meals	0.6818	0.7667	5.7629	0.0981	4	0.6794	337	0.6217
EAT_26	Eating	Enjoy trying new rich foods	0.7692	0.8500	8.4015	0.1313	6	0.7178	618	0.8319
BSQ_1	Body shape	Worried about shape	0.7595	0.8417	9.1916	0.1260	5	0.6878	564	0.7986
BSQ_2	Body shape	Compare body shape	0.8696	0.9250	12.9378	0.1578	5	0.7082	903	1.0000
BSQ_3	Body shape	Felt fat when naked	0.7595	0.8417	8.8589	0.1264	5	0.6976	572	0.8010
BSQ_4	Body shape	Avoid social situations	0.7792	0.8583	9.3847	0.1337	5	0.7021	634	0.8476
BSQ_5	Body shape	Worried others seeing fat	0.7692	0.8500	9.8545	0.1290	5	0.6841	589	0.8175
BSQ_6	Body shape	Worried taking up much space	0.8000	0.8750	10.7493	0.1385	5	0.6949	688	0.8781
BSQ_7	Body shape	Check fat levels	0.7500	0.8333	9.2582	0.1219	5	0.6744	526	0.7725
BSQ_8	Body shape	Avoid situations	0.7407	0.8250	8.2751	0.1200	5	0.6910	512	0.7605
IUS_1	Uncertainty	Stops having a firm opinion	0.7895	0.8667	11.0030	0.1343	6	0.6839	647	0.8513
IUS_2	Uncertainty	Disorganized	0.7500	0.8333	9.0636	0.1219	6	0.6782	529	0.7726
IUS_3	Uncertainty	Intolerable	0.7317	0.8167	7.2827	0.1176	6	0.7041	495	0.7455
IUS_4	Uncertainty	Unfair not having guarantees	0.8108	0.8833	12.1913	0.1399	6	0.6783	702	0.8867
IUS_5	Uncertainty	Mind unrelaxed	0.7595	0.8417	9.3136	0.1254	6	0.6902	566	0.7949
IUS_6	Uncertainty	Uneasy, anxious, or stressed	0.7500	0.8333	8.2058	0.1234	6	0.7064	551	0.7825
IUS_7	Uncertainty	Unforeseen events upset	0.7792	0.8583	9.4305	0.1330	6	0.7043	636	0.8429
IUS_8	Uncertainty	Frustation for not having information	0.8219	0.8917	13.1027	0.1425	6	0.6744	729	0.9030
IUS_9	Uncertainty	Keeps me from living a full life	0.7692	0.8500	9.6244	0.1289	5	0.6899	594	0.8169
IUS_10	Uncertainty	Avoid surprises	0.7500	0.8333	8.6831	0.1231	6	0.6910	539	0.7803
IUS_11	Uncertainty	Unforeseen event can spoil	0.7059	0.7917	6.6205	0.1080	6	0.6840	407	0.6845
IUS_12	Uncertainty	Uncertainty paralyses me	0.7895	0.8667	11.5370	0.1331	6	0.6734	637	0.8439
IUS_13	Uncertainty	I am not first rate	0.7407	0.8250	8.7615	0.1187	6	0.6775	502	0.7526
IUS_14	Uncertainty	I can’t go forward	0.7895	0.8667	10.8928	0.1350	6	0.6829	646	0.8560
IUS_15	Uncertainty	I can’t function very well	0.7792	0.8583	9.8419	0.1326	6	0.6966	629	0.8405

Label	Dimension	ID	Closeness centrality	Harmonic closeness centrality	Betweeness centrality	Authority	Modularity	Clustering. coefficient	Number triangles	Eigenvector centrality
IUS_16	Uncertainty	Others know where they are going	0.8000	0.8750	11.5336	0.1372	6	0.6828	676	0.8698
IUS_17	Uncertainty	Vulnerable, unhappy, or sad	0.7792	0.8583	10.1697	0.1317	6	0.6910	624	0.8347
IUS_18	Uncertainty	I want to know what the future	0.7895	0.8667	10.2412	0.1352	6	0.6977	660	0.8572
IUS_19	Uncertainty	I can't stand being taken by surprise	0.7895	0.8667	10.3159	0.1358	6	0.6987	661	0.8605
IUS_20	Uncertainty	Smallest doubt can stop me	0.6742	0.7583	5.4511	0.0941	6	0.6796	316	0.5970
IUS_21	Uncertainty	Organize everything in advance	0.8108	0.8833	11.3520	0.1415	6	0.6957	720	0.8971
IUS_22	Uncertainty	I lack confidence	0.7692	0.8500	9.4707	0.1286	6	0.6934	597	0.8154
IUS_23	Uncertainty	Unfair other seem sure about future	0.7407	0.8250	7.6797	0.1210	6	0.7031	521	0.7670
IUS_24	Uncertainty	Don't sleep soundly	0.7059	0.7917	7.1422	0.1063	6	0.6756	402	0.6741
IUS_25	Uncertainty	Get away from uncertain situations	0.8000	0.8750	10.3318	0.1389	6	0.7040	697	0.8802
IUS_26	Uncertainty	Ambiguities stress me	0.7059	0.7917	6.8260	0.1067	6	0.6824	406	0.6763
IUS_27	Uncertainty	I can't stand being undecided	0.7792	0.8583	10.1690	0.1316	6	0.6888	622	0.8341

Figure S1 *Centrality analysis stability plot and edge-weight accuracy plot within the clinical subsample*

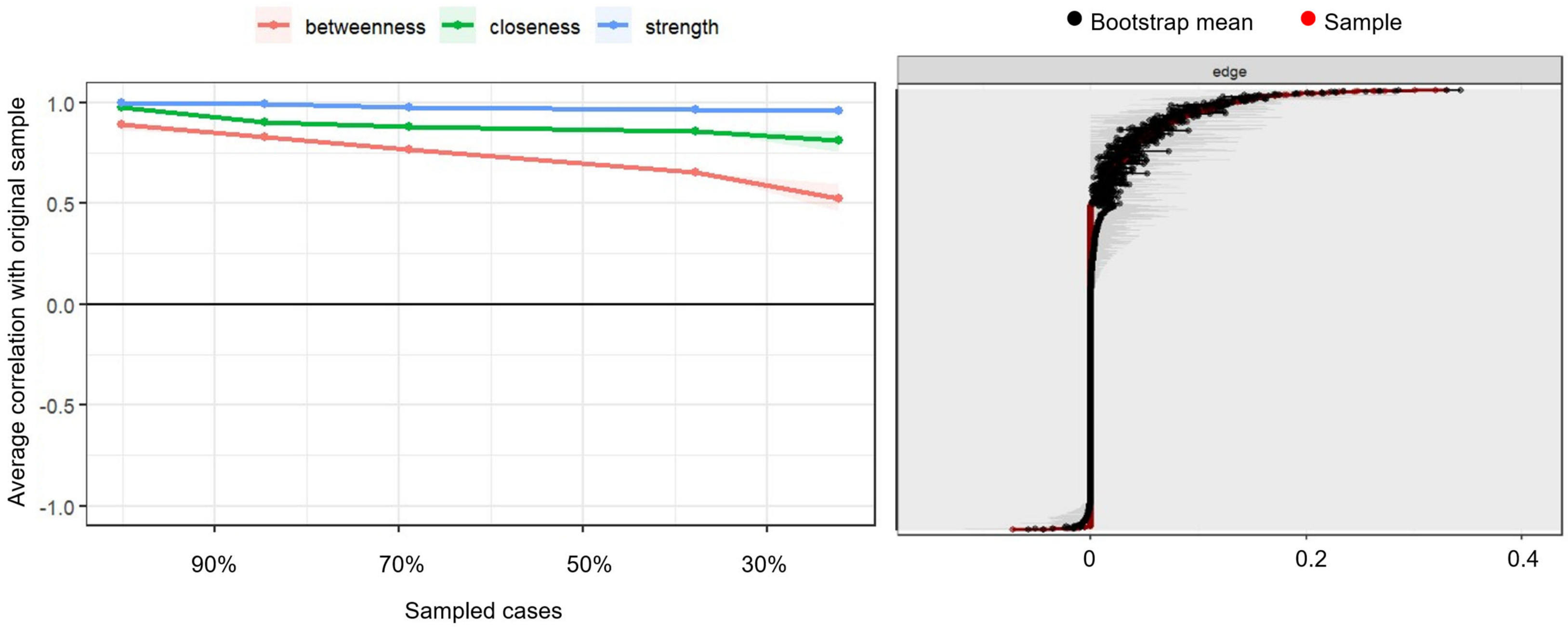

Figure S2 *Centrality analysis stability plot and edge-weight accuracy plot within the control subsample*

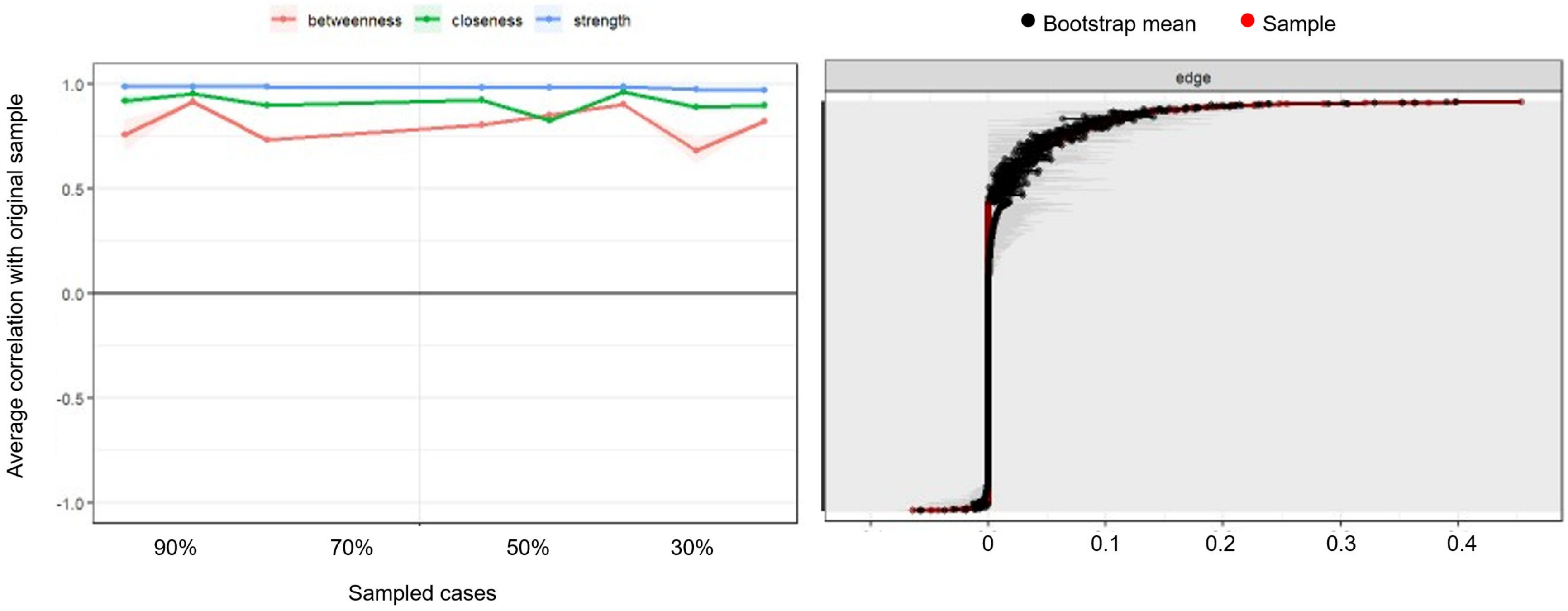

Supplement: Supplementary file 1 [file nutrients-18-01370-s001.zip › nutrients-4233008-supplementary.pdf]
